# MYB Transcription Factor Family in Pearl Millet: Genome-Wide Identification, Evolutionary Progression and Expression Analysis under Abiotic Stress and Phytohormone Treatments

**DOI:** 10.3390/plants12020355

**Published:** 2023-01-12

**Authors:** Jeky Chanwala, Badrinath Khadanga, Deepak Kumar Jha, Inavolu Sriram Sandeep, Nrisingha Dey

**Affiliations:** 1 Division of Plant and Microbial Biotechnology, Institute of Life Sciences, NALCO Nagar Road, NALCO Square, Chandrasekharpur, Bhubaneswar 751023, India; 2 Regional Centre for Biotechnology, Faridabad 121001, India

**Keywords:** MYB transcription factors, evolutionary progression, pearl millet, phytohormones, abiotic stress

## Abstract

Transcription factors (TFs) are the regulatory proteins that act as molecular switches in controlling stress-responsive gene expression. Among them, the MYB transcription factor family is one of the largest TF family in plants, playing a significant role in plant growth, development, phytohormone signaling and stress-responsive processes. Pearl millet (*Pennisetum glaucum* L.) is one of the most important C4 crop plants of the arid and semi-arid regions of Africa and Southeast Asia for sustaining food and fodder production. To explore the evolutionary mechanism and functional diversity of the MYB family in pearl millet, we conducted a comprehensive genome-wide survey and identified 279 MYB TFs (PgMYB) in pearl millet, distributed unevenly across seven chromosomes of pearl millet. A phylogenetic analysis of the identified PgMYBs classified them into 18 subgroups, and members of the same group showed a similar gene structure and conserved motif/s pattern. Further, duplication events were identified in pearl millet that indicated towards evolutionary progression and expansion of the MYB family. Transcriptome data and relative expression analysis by qRT-PCR identified differentially expressed candidate *PgMYBs* (*PgMYB2*, *PgMYB9*, *PgMYB88* and *PgMYB151*) under dehydration, salinity, heat stress and phytohormone (ABA, SA and MeJA) treatment. Taken together, this study provides valuable information for a prospective functional characterization of the MYB family members of pearl millet and their application in the genetic improvement of crop plants.

## 1. Introduction

Environmental stresses and climate change pose a serious threat to global agricultural productivity. Plants face multiple stresses that challenge their growth and survival [1,2]. As a result, plants respond to these stress conditions, by activating various signaling pathways and by the synthesis of specialized metabolites. These responses are regulated by various transcription factor-encoding genes [3]. Transcription factors (TFs) bind to their cognate sites in the promoter region of the target gene and regulate the gene expression. Upon environmental stress on plants, TFs either induce or repress the expression of target genes [4,5]. Understanding the involvement of particular TF in various stress-signaling pathways will be helpful in developing stress-resistant and genetically modified (GM) crop plants with enhanced productivity. In recent times, several studies have identified and characterized various TF gene families, such as bZIP (basic leucine zipper), WRKY, MYB (v-myb avian myeloblastosis viral oncogene homolog), NAC, bHLH (basic helix-loop-helix), GRAS (Gibberellic-acid insensitive, repressor of GAI, and Scarecrow), THX (Trihelix) and ERF (Ethylene response factor), which are known to participate in multiple stresses and involved in phytohormonal signaling pathways [6,7].

Rising world population challenges food and nutrition security across the globe [8]. In search for suitable, staple foods for overcoming such difficult scenarios, millets are one of the potential candidates. Among them, pearl millet (*Pennisetum glaucum* L.) is a monocot plant under the grass family (*Poaceae*), which is considered on sixth position in terms of global food importance after rice, wheat, maize, barley and sorghum. It is an annual cereal crop species, cultivated in arid and semi-arid tropical regions of Africa and Southeast Asia, including India [9]. It has the potential to grow in adverse climatic conditions such as high temperature, drought and less fertile soil. In addition, there are various nutritional aspects of pearl millet, rich in protein, carbohydrate and fiber content. In addition, pearl millet also contains vitamins and micronutrients such as zinc, iron and magnesium [10]. The IPMGSC (International Pearl Millet Genome Sequencing Consortium) has sequenced the whole genome of pearl millet [11] to improve crop production. Further studies on gene family identification and characterization will be helpful in identifying the stress-associated genes and other TFs.

The MYB gene family is one of the major TF family found in both plants and animals. The discovery of the first MYB gene was an oncogene found in the avian myeloblastosis virus known as ‘ʋ-MYB’. The MYB protein has a conserved signature structure, i.e., a DNA-binding module of MYB TF with regularly interspaced three tryptophan residues to form a 3D helix-turn-helix structure which comprises 52 amino acids. Based on the adjacent repeats, MYB TFs are classified into four groups, viz. 1R-MYB, 2R-MYB (R2R3-MYB), 3R-MYB (R1R2R3-MYB) and 4R-MYB (R1R2R2R1/2). In plants, generally R2R3-MYB-type proteins are seen and studies have shown that the R2R3-MYB genes evolved from R1R2R3-MYB. To date, MYB TF members have been identified in various plant species such as rice (*Oryza sativa; OsMYB*), wheat (*Triticum aestivum* L.; *TaMYB*), maize (*Zea mays*; *ZmMYB*), Arabidopsis (*Arabidopsis thaliana*; *AtMYB*), soybean (*Glycine max*; *GmMYB)*, etc. [12,13,14]. Being a large and diverse family, MYB TF plays a vital role in biotic/abiotic stress responses, plant growth, seed and flower development, cell cycle regulation, secondary metabolites synthesis and hormonal signaling [15]. An overexpression of *AtMYB44* and *OsMYB4* conferred a tolerance to abiotic stress in Arabidopsis and rice, respectively [16,17,18]. Similarly, overexpression of *TaMYB344* in tobacco resulted in enhanced resistance of the plants to dehydration, heat and salinity stress [19]. Tang et al. 2019, reported the role of *OsMYB6* under drought and salt stress. It was seen that *OsMYB6* transgenic plants showed an enhanced tolerance compared to wild plants [20].

Phytohormones play a crucial role in various physiological processes, as well as in mediating responses against biotic and abiotic stresses [21]. The levels of signal molecules, such as calcium, jasmonic acid, methyl jasmonate and salicylic acid, are seen to be altered in plants encountering stresses [22]. Abscisic acid (ABA) plays a vital role in plant physiological processes and is an important mediator in environmental stresses such as drought, cold, light, salinity and temperature [23]. The jasmonate signal molecules (methyl jasmonate (MeJA) and jasmonic acid (JA)) are lipid-derived molecules and play key roles in plant biotic stress responses and other biological processes. Several studies showed the upregulation of JA biosynthetic genes in roots under salt stress [24,25]. Similarly, the signaling molecule, salicylic acid, also regulates plant metabolism and is crucial for induction of stress responses in plants [26,27].

Though MYB TF has been identified in various plant species, there are no reports on the identification and functional analysis of the MYB family in pearl millet (*P. glaucum*). Considering the relevance, this nascent study aims for a genome-wide identification of the MYB TFs in pearl millet. In silico characterization and evolutionary progression of the identified MYB family members were performed through an analysis of the motif composition, gene structure, phylogeny and synteny relationship. Additionally, transcriptome profiling and the relative expression profile of selected *PgMYBs* were evaluated under dehydration, salinity, heat-stress and phytohormone (ABA, SA and MeJA) treatments, with an aim to identify stress-inducible MYB TFs. Further, the candidate *PgMYBs* could appear as potential tools for developing engineered crop plants to ensure better crop productivity.

## 2. Materials and Methods

### 2.1. Identification and Sequence Analysis of MYB TFs in Pearl Millet

Pearl millet protein and nucleotide sequences were downloaded from the genome database of pearl millet (http://cegsb.icrisat.org/ipmgsc/ (accessed on 23 May 2019)). In addition, publicly available transcriptome datasets on pearl millet were also used to identify the complete repertoire of MYB family members in pearl millet [28,29,30]. The MYB family protein sequences of rice were downloaded from Oryzabase (http://rice.plantbiology.msu.edu/ (accessed on 18 February 2020) [31] and GRASSIUS (Grass Regulatory Information Server) (accessed on 18 February 2020) [32]. Arabidopsis and foxtail millet MYB domain protein sequences were obtained from the plant genomics database, Phytozome [33]. The identification of MYB in pearl millet was initiated by constructing a hidden Markov model (HMM) profile of the reference sequences (rice, Arabidopsis and foxtail millet) through the hmmbuild program of the HMMER tool v3.2 [34], followed by a hmmsearch against pearl millet proteome. The initially identified sequences were then scanned against the HMM profiles obtained from the Pfam site [35]. All the non-redundant hits with an expected value cut-off of (0.01) were retained and the redundant sequences were removed. Further, the putative MYB genes were confirmed for the presence of MYB by using hmmscan (https://www.ebi.ac.uk/Tools/hmmer/search/hmmscan (accessed on 4 March 2020), SMART (Simple Modular Architecture Research Tool) (http://smart.embl-heidelberg.de/ (accessed on 4 March 2020)) and CDD (Conserved Domains Database) (http://www.ncbi.nlm.nih.gov/Structure/cdd/wrpsb.cgi (accessed on 4 March 2020).

The prediction of the theoretical isoelectric point, protein molecular weight, instability index, GRAVY and aliphatic index for each protein was checked by using the ProtParam tool (https://web.expasy.org/protparam/ (accessed on 2 April 2020). Further, the TMHMM—2.0 tool [36] was employed for the location of transmembrane helices in the PgMYB sequences. WoLF PSORT [37] was used for predicting subcellular localization sites in PgMYB sequences.

### 2.2. Chromosomal Localization, Gene Structure and Motif Analysis

The PgMYBs genes were mapped to the pearl millet chromosomes according to their physical positions (bp) with the help of MapInspect software v1.0 (http://mapinspect.software informer.com/ (accessed on 01st May 2020). The exon/intron structures of each *MYB* gene in the pearl millet were determined by the GSDS (Gene Structure Display Server) tool (accessed on 26 April 2020) [38]. In order to identify the conserved motifs in PgMYB sequences, the MEME suite (accessed on 18 April 2020) [39] was used with the following parameters: (20; maximum number of motifs, 6; minimum width and 50; maximum width). Further, the results obtained were visualized by TBtools [40].

### 2.3. Phylogenetic Analysis, Gene Duplication and Synteny Analysis

For the phylogenetic analysis, the MYB sequences of pearl millet, rice, *Arabidopsis* and foxtail millet were used. A multiple sequence alignment was done using MUSCLE with the default parameters [41]. The phylogenetic tree was constructed by the MEGA V7.0 (Molecular Evolutionary Genetics Analysis) program [42] using the neighbor-joining method (with 1000 bootstrap replicates), with the following parameters (Jones–Taylor–Thronton model; rates among sites: gamma distributed (G) and partial deletion of gaps). The *MYB* gene-duplication events were examined by Multiple Collinearity Scan toolkit (MCScanX) with the default parameters [43]. The synteny relationship between the *PgMYB* genes and the *MYB* genes from *Oryza sativa*, *Arabidopsis thaliana and Setaria italica* were visualized by AccuSyn software [44].

### 2.4. Cis-Acting Regulatory Elements Analysis (CREs)

The promoter sequences (2000 bp upstream region from translation start site) of all the identified *PgMYB* genes were extracted from the pearl millet genome database. The sequences were uploaded into the PlantCARE server (accessed on 23 April 2020) [45] for identifying the cis-acting regulatory elements present in the promoter region of the identified *PgMYBs.*

### 2.5. In Silico Expression Analysis

For investigation of the expression profiles of the PgMYB genes, transcriptome datasets of pearl millet under drought and salinity stress (SRX3556461, SRX3556459, SRX6918725, SRX6918726) were downloaded from the SRA database of NCBI (https://www.ncbi.nlm.nih.gov/sra (23 February 2021)) [29,30]. First, the transcriptome datasets were aligned and mapped using Bowtie 2.0 tool, and the RSEM (RNA-Seq by Expectation-Maximization) software was used to quantify the RNA-seq reads [46,47,48]. Further, the differentially expressed genes were identified and MATRIX file was used to generate a heatmap using TBtools software.

### 2.6. Plant Growth and Stress Treatments

Pearl millet seeds PRLT 2/89-33 were obtained from the International Crops Research Institute for Semi-Arid Tropics (ICRISAT) through a material transfer agreement (MTA). The seeds were sown in nutrient soil (1:1 mix of black and red soil) and allowed to grow in a greenhouse with a 16:8 h light:dark cycle at 28 °C (±2).

Four-week-old pearl millet seedlings were subjected to a drought condition by withholding water for 8 days, whereas the control plants were watered on alternate days. On the 9th day, both the control and treated plants were rewatered for their recovery. After treatment, well-expanded leaf samples of both the control and treated plants were collected on 0, 5, 7, 9 and 11 days, respectively. For salt stress, four-week-old seedlings were transferred to Hoagland solution containing 250 mM NaCl and, for the control, ½ strength Hoagland solution was used. Leaf samples were collected from both the control and treated plants at time points of 0 h, 3 h (early) and 24 h (late). For heat stress, plants were transferred to a 45 °C chamber for 12 h and leaf samples were collected at 0, 3, 12 and 24 h time points [28,49].

For the hormonal stress experiments, four-week-old seedlings were treated with 100 µM Abscisic acid (ABA), 100 µM Salicylic acid (SA) and 100 µM methyl jasmonate (MeJA) [50]. Leaf samples from both the control and treated plants were collected at different time points of 0 h, 3 h (early) and 24 h (late). The tissue-specific expression of the *PgMYB* genes was also studied by harvesting the leaf, stem and root from four-week-old plants under normal conditions. The harvested samples were snap-frozen in liquid nitrogen and stored at −80 °C until further analysis. All the samples were collected in biological triplicates.

### 2.7. Quantitative qRT-PCR Analysis

Total RNA was isolated from the samples by using a Spectrum Plant Total RNA Kit (Sigma-Aldrich, Lt. Louis, MO, USA) according to manufacturer’s instructions. The RNA quality was checked on a 1.2% agarose gel with 18% formaldehyde. The purity and yield were estimated by a NanoDrop ND-2000 spectrophotometer (Thermo Scientific, Waltham, MA, USA) and RNA samples with a 260/280 nm ratio from 2.0 to 2.1 were used for further analysis. RNA purification was done by treating with DNAse I (Sigma-Aldrich, MO, USA) as per the manufacturer’s protocol.

A cDNA synthesis was carried out by a First Strand cDNA Synthesis Kit (Thermo Scientific, Waltham, MA, USA). An expression analysis of selected *MYB* genes was performed using the QuantStudio™ 3 Real-Time PCR System (Applied Biosystems, Foster City, CA, USA). All the primers used in this study were designed by the PrimerQuest tool of IDT. The qRT-PCR reaction mixture included a total volume of 20 μL containing 2 μL (20 ng) of cDNA, 10 μL of SYBR premix buffer (Mesa Green qPCR Master Mix (Eurogentec)), 1.0 μL each of forward and reverse primers (5 μM) and 6 μL of nuclease-free water. The qRT-PCR run profile was as follows: 95 °C for 10 min, followed by 40 cycles of 95 °C for 15 s, and 60 °C for 1 min. EF1α (elongation factor 1 alpha) and *GAPDH* (glyceraldehyde 3-phosphatedehydrogenase) [51] genes were taken for data normalization.

## 3. Results and Discussion

### 3.1. Identification and Physicochemical Characteristics of MYB TF Family in Pearl Millet

The HMM tool was used for the screening of MYB TF family members in the pearl millet. A total of 306 probable MYB members were identified using hmmsearch. All the redundant sequences were removed using the Expasy tool and the MYB DNA-binding domain (PF00249) was confirmed in the non-redundant putative MYB sequences using the hmmscan, SMART and CDD tools. Finally, a total of 279 MYB TFs were identified in the pearl millet and designated as PgMYB1 to PgMYB279, based on their chromosomal location and coordinates. Interestingly, the identified MYB members in pearl millet were higher in number compared to that of *Arabidopsis* (155), *O. Sativa* (197) *S. bicolor* (134), *Soyabean* (244), *S. italica* (209) and *Z. maize* (157) [52,53,54,55,56].

The physical and chemical properties, such as protein length, molecular weight (MWs), isoelectric point (PI), and subcellular localization of the identified PgMYBs, were analyzed (Additional Table S1). The amino acid length of the PgMYBs varies from 73 (PgMYB190) to 2299 (PgMYB250) amino acids and the relative molecular weight ranges from 7.46 kDa (PgMYB190) to 255.88 kDa (PgMYB250). Moreover, the theoretical isoelectric point (pI) of the identified PgMYB proteins ranges from 4.37 to 11.34, and the grand average of hydropathy (GRAVY) value of all the PgMYB proteins was found to be relatively low (<0), which suggests their hydrophilic nature. The prediction of their subcellular localization showed that most of the PgMYB proteins (234 PgMYBs) were located in the nucleus, which suggests their role as a TF. However, some PgMYB were localized in the chloroplast (23), cytoplasm (11), mitochondria (7), peroxisome (1) and plasma membrane (1). Further, PgMYB66 and PgMYB152 were predicted to contain transmembrane (TM) helices by the TMHMM server. (Additional Figure S1). Membrane-bound TFs (MTFs) are known to play a significant role in biotic and abiotic stress responses. The MTFs get activated by proteolytic cleavage during environmental stresses [57,58].

### 3.2. Chromosomal Localization, Gene Structure and Motif Analysis of PgMYBs

**Figure 1:**
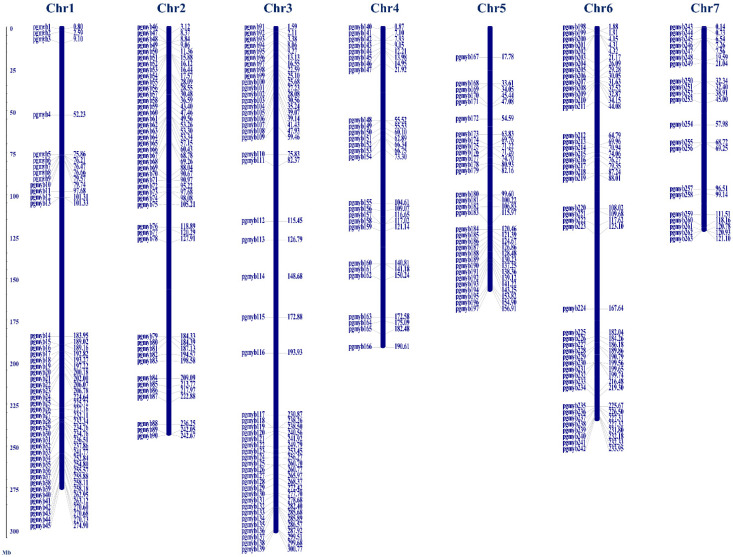
Chromosomal mapping of identified 279 PgMYB members on pearl millet genome.

The pearl millet genome comprises seven chromosomes with varying lengths: chromosome 3 being the longest (300.9 Mb), and chromosome 5 being the shortest (158.6 Mb). The identified 279 PgMYBs were mapped on all the chromosomes based on their positions and chromosomal coordinates in pearl millet genome using MapInspect software v1.0. The physical map positions demonstrated that the PgMYBs were unevenly distributed across all seven chromosomes (Figure 1). The maximum number of *PgMYB* genes were located on chromosome 3 (49 *PgMYBs*), followed by chromosomes 1, 2 and 6 with 45 *PgMYBs* individually. Comparatively, fewer *PgMYBs* were located on chromosomes 5 (31) and 7 (21). The larger number of *PgMYBs* on chromosomes 3, 1, 2 and 6 indicate a possible hot spot region for MYB family member’s distribution during the course of pearl millet evolution.

The diversification of gene structure is an important component of gene family evolution and also contributes to phylogenetic groupings. The exon/intron organization was analyzed to gain insights into the structural variation of *PgMYB* genes by the GSDS server. The number of introns in the *PgMYB* genes varied from 0 to 23; however, 21 *PgMYBs* did not contain an intron, while 56 *PgMYBs* had only one intron (Additional Figure S2). The maximum number of introns were observed in *PgMYB264* (23), followed by *PgMYB223* with 21 introns. The results indicate a high structural diversity of the *PgMYB* genes in pearl millet.

**Figure 2:**
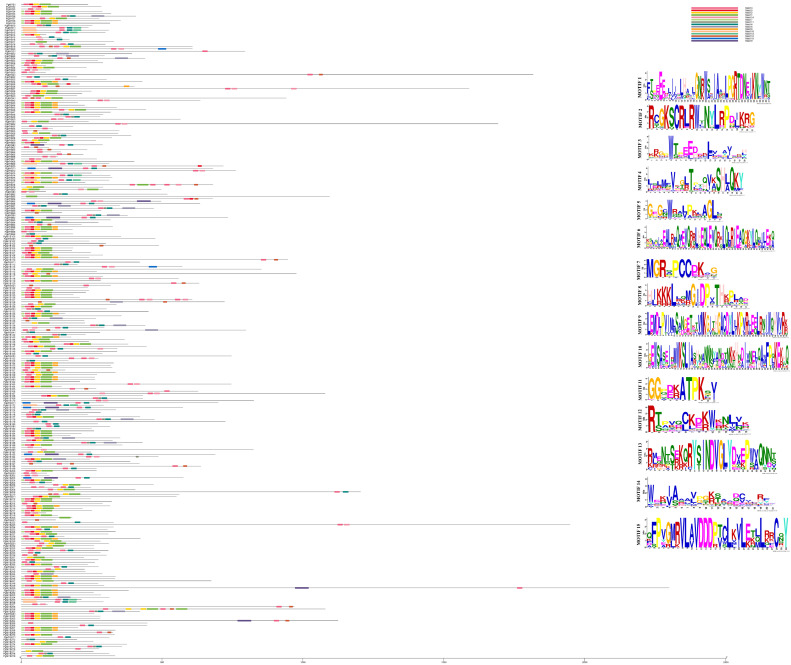
Conserved motif distribution in identified 279 PgMYB proteins. All identified 15 motifs are indicated by different colors and their respective sequence logo.

A total of 15 distinct conserved motifs were identified in the PgMYBs (Figure 2). Motif 1, Motif 2, Motif 4 and Motif 5 were present in most of the PgMYBs, with conserved residues that are identical among all the R2R3-MYB domains, while Motif 3 was found to be a core conserved motif in the identified PgMYBs. Along with the typically conserved residue Trp (W), other residues such as Gly (G), Glu (E), Asp (D), Cys (C), Arg (R), Leu (L), Thr (T), Asn (N) and Lys (K) were also conserved. However, a few motifs such as Motif 6, Motif 9, Motif 10, Motif 13 and Motif 15 were present in less than 10% of the PgMYB sequences. We also observed Motif 1, Motif 2, Motif 3, Motif 5 and Motif 7 were located towards the N-terminal, while Motif 4 and Motif 6 were found towards the C-terminal. Most of the PgMYB members from the same clade of the phylogenetic tree displayed similar motif compositions, which indicates their functional similarities within the same subgroup.

### 3.3. Phylogenetic Relationship and Gene Duplication Events of PgMYBs

**Figure 3:**
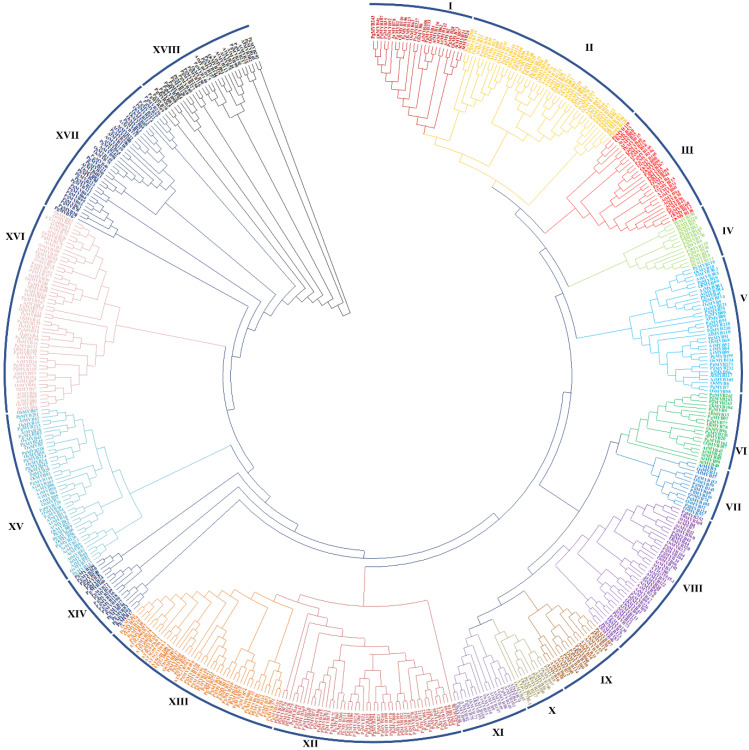
Phylogenetic tree of MYB family members of *P. glaucum*, *A. thaliana* and *O. sativa.* A total of 534 MYB proteins were aligned by Clustal, and tree was constructed by MEGA v7.0 using maximum likelihood method with 1000 bootstrap replication. The different groups are numbered from (I–XVIII).

To explore the evolutionary relationships among the 279 PgMYBs, 126 OsMYBs and 141 AtMYBs, a phylogenetic tree was constructed by using MEGA7 software. We perceived from the phylogenetic tree (Figure 3) that most of the *PgMYBs* were aligned in 18 (Group I-XVIII) subgroups with *OsMYB* and *AtMYB*. Among the 18 groups, Group XII found to be the largest, with 47 PgMYBs, followed by Group XIII (34 PgMYBs), Group XVII (22 PgMYBs), Group II (21 PgMYBs), Group XVIII (20 PgMYBs), Group XVI (19 PgMYBs), Group XIV (17 PgMYBs), Group XV (15 PgMYBs), Group V (14 PgMYBs), Group IV (13 PgMYBs), Group VIII (12 PgMYBs), Group III (11 PgMYBs), Group VI (8 PgMYBs), Group I (8 PgMYBs), Group VII (6 PgMYBs), Group IX (5 PgMYBs), Group XI (4 PgMYBs), and Group X was found to be smallest with 3 PgMYBs.

**Figure 4:**
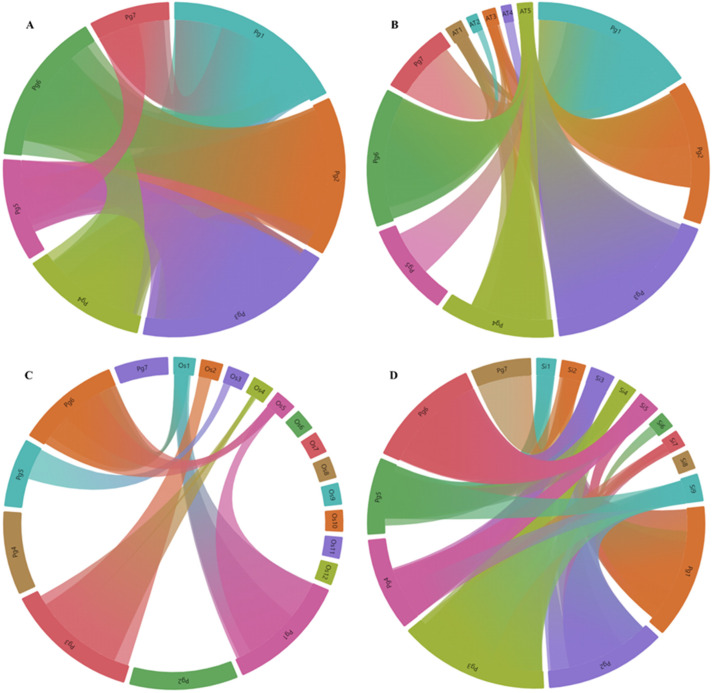
Synteny relationship of MYB family members between *P. glaucum*, *A. thaliana*, *O. sativa* and *S. italica*. Each connected colored line shows orthologous and paralogous pairs among the species. (**A**) Paralogous relationship in *P. glaucum* (**B**) Orthologous relationship between *P. glaucum* and *A. thaliana* (**C**) Synteny relationship between *P. glaucum* and *O. sativa* (**D**) Synteny relationship in *P. glaucum* and *S. italica*.

Further, we performed a synteny analysis to identify the duplication events among the MYB family members of pearl millet, rice and foxtail millet using the MCScanX tool. We observed both orthologous and paralogous events across all seven chromosomes of the pearl millet. Moreover, the PgMYBs from chromosome 1, chromosome 3 and chromosome 6 of the pearl millet were predominantly involved in an orthologous relationship. A total of 198 paralogous pairs, including 22 tandem and 176 segmental events, were identified in the MYB family members of the pearl millet (Figure 4).

A total of 204 *PgMYBs* were showing an orthologous relationship with the MYB family members of Arabidopsis, rice and foxtail millet. Foxtail millet showed the highest collinear relationship (70%) with 343 collinear genes, followed by Arabidopsis (48%) with 205 collinear genes and rice (28%) with 111 collinear genes (Additional Table S2). Interestingly, we found 9 collinear *PgMYB* pairs with rice and foxtail millet but not with Arabidopsis, indicating their probable formation after the monocot and dicot divergence, whereas, 14 collinear pairs of *PgMYB* occurred with Arabidopsis, but not with rice and foxtail millet, which suggests their probable disappearance during the divergence of monocot and dicot. Similarly, nine collinear *PgMYB* pairs were found only with foxtail millet, but not with Arabidopsis and rice, which suggests these pairs may be formed during the millets’ evolution; while six collinear *PgMYB* pairs were found with Arabidopsis and rice, but not with foxtail millet, which suggests their loss during the millets’ evolution. The formation and loss of collinear pairs shows the evolutionary progression of the MYB family members in pearl millet [59,60].

### 3.4. Cis-Acting Regulatory Elements Analysis in Promoter Regions of PgMYBs

**Figure 5:**
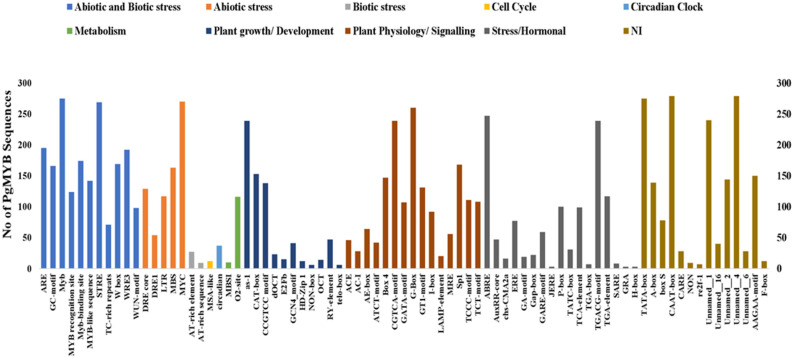
Presence of cis-acting regulatory elements in 2000 bp upstream region of identified PgMYBs. Color of bars indicates the group (shown in legend) to which it belongs.

The cis-acting regulatory elements’ analysis of promoters helps in understanding gene regulation at the transcriptional level. Therefore, the putative cis-acting regulatory elements present in the 2000 bp upstream region of the identified PgMYBs were identified using the PlantCARE database. The results demonstrated the presence of versatile cis-acting regulatory elements (Figure 5) linked to plant growth and development (CAT-box, dOCT, E2Fb, HD-Zip, OCT, etc.), hormonal signaling (AuxRR, ERE, JERE, GA-motif, GARE-motif, P-box, etc.), circadian cycle, metabolism (O_2_-site), cell cycle regulation (MSA-like), seed-specific regulation (RY element), XYLEM specific expression (AC-I & II) and abiotic/biotic stress (ARE, ABRE, DRE, LTRMYB, W-box, WUN-motif, MBS, etc.,) responses. The presence of versatile cis-acting regulatory elements in the presumptive promoter regions of the *PgMYBs* shows their functional diversity and their probable involvement in multiple biological plant processes [61,62].

### 3.5. In Silico Expression Analysis of PgMYBs under Dehydration and Salinity Stress

The RNA-seq data was analyzed to assess the expression level of the identified 279 *PgMYB* genes under dehydration and salinity stress conditions in pearl millet. With respect to this, the publicly available transcriptome data and the Sequence Read Archive (SRA-NCBI) files were accessed and explored for the differential expression profiling of *PgMYBs*. As shown in Additional Figure S3a,b, most of the *PgMYBs* showed differential transcripts level under both dehydration and salinity stress. Specifically, upon dehydration stress, *PgMYB2*, *PgMYB9*, *PgMYB61*, *PgMYB8*, *PgMYB106*, *PgMYB110*, *PgMYB114*, *PgMYB169*, *PgMYB198*, *PgMYB250*, *PgMYB269 and PgMYB275* showed a higher expression level, whereas the expression level of *PgMYB138*, *PgMYB141*, *PgMYB228*, *PgMYB229*, *PgMYB245* and *PgMYB246* was decreased. Similarly, under salinity stress, the expression level of *PgMYB2*, *PgMYB9*, *PgMYB35*, *PgMYB49*, *PgMYB101*, *PgMYB102*, *PgMYB134*, *PgMYB146*, *PgMYB151*, *PgMYB241*, *PgMYB249*, *PgMYB267* and *PgMYB271* was induced, while the transcripts accumulation for *PgMYB126*, *PgMYB199*, *PgMYB201*, *PgMYB235* and *PgMYB240* was reduced as compared to the control samples.

We tried to correlate the expression pattern of *PgMYBs* in dehydration and salinity stress. Here, we found similar upregulated expression profiles of *PgMYB2* and *PgMYB9* under both dehydration and salinity stresses, which suggests their probable involvement in abiotic stress responses. We also observed a few *PgMYBs* of the same phylogenetic clade or group showed a similar expression pattern under both dehydration and salinity stress, which suggests the similar functional profiling of subfamily members [15].

### 3.6. Relative Expression Analysis of PgMYBs

Previous studies have shown the role of *MYB* family members in regulating environmental stress responses [15]. We have selected 15 *PgMYB* genes (Additional Table S3) based on in silico expression profiling, phylogenetic analysis, sequence homology and synteny analysis to explore their expression profiling in different tissues and under abiotic stress conditions.

### 3.7. Tissue-Specific Expression Profiling of PgMYBs

Tissue-specific expression analysis helps in understanding the role of a particular TF/gene in the growth and development of the plant. Therefore, we evaluated the spatial expression pattern of selected 15 *PgMYBs* in the leaf, stem and root tissues of pearl millet. As shown in Additional Figure S4, the transcript level of *PgMYB9*, *PgMYB44*, *PgMYB61*, *PgMYB88*, *PgMYB132*, *PgMYB151* and *PgMYB198* was predominant in the leaf tissues, whereas the expression level of *PgMYB49*, *PgMYB187* and *PgMYB263* was significantly higher in the root tissues compared to the leaf and stem tissues. We also observed higher expression of four *PgMYBs,* namely *PgMYB2*, *PgMYB134*, *PgMYB176* and *PgMYB240,* in both the leaf and root tissues. *PgMYB229* was only expressed in stem tissues, predominantly. Taken together, the tissue-specific expression profiling of *PgMYBs* provides a basis for better understanding of pearl millet growth and development [63].

### 3.8. Relative Expression Analysis of PgMYBs under Abiotic Stress

To investigate the role of *PgMYB* genes under different abiotic stresses in pearl millet, the expression profile of 15 selected *PgMYB* genes was generated at different time points under dehydration, salinity and heat stress.

**Figure 6:**
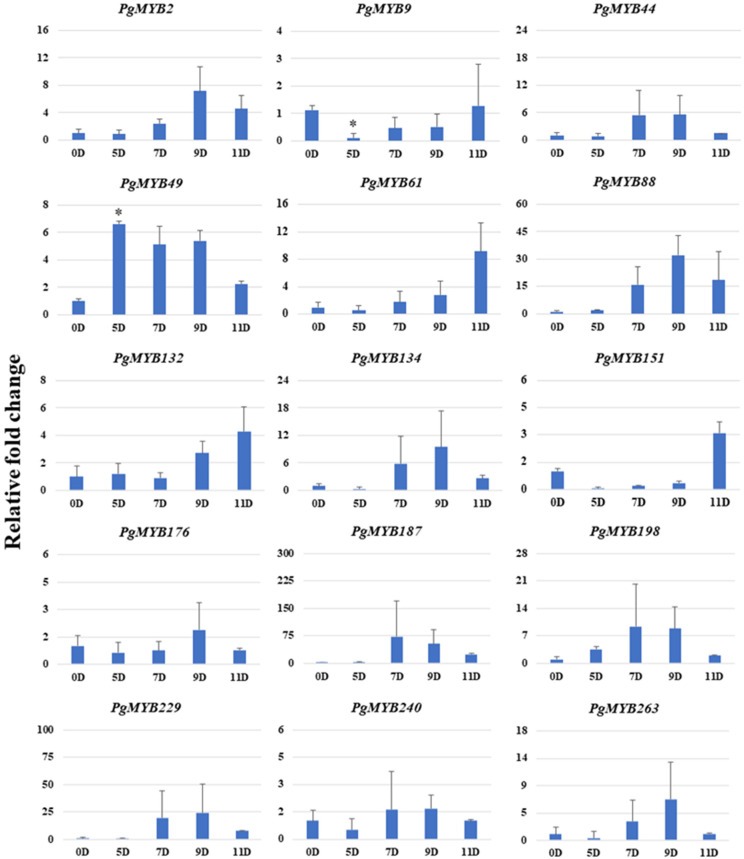
Relative expression analysis of *PgMYB* genes under dehydration stress at 0th day, 5th day, 7th day, 9th day and 11th day time points. The *X*-axis represents different time points and the *Y*-axis indicates relative expression level. Significant difference in mean indicated by * *p* < 0.05 as obtained by Student’s *t*-test.

The *PgMYBs* showed a differential expression pattern under the dehydration stress condition (Figure 6). Among the 15 selected *PgMYBs*, 13 *PgMYB* genes showed an upregulation pattern upon dehydration stress, whereas only 2 *PgMYBs* showed a downregulated expression pattern. Moreover, *PgMYB2*, *PgMYB49 and PgMYB88* were significantly induced, while the expression level of *PgMYB151* was prominently downregulated. Interestingly, we observed a recovery in the transcripts level of *PgMYB44*, *PgMYB134*, *PgMYB151*, *PgMYB187* and *PgMYB198* on 11th day (after rewatering). This suggests their possible involvement in the dehydration stress responses of pearl millet. We also noticed that qRT-PCR analysis data of most of the *PgMYBs* corroborated with the transcriptome profile under dehydration stress.

**Figure 7:**
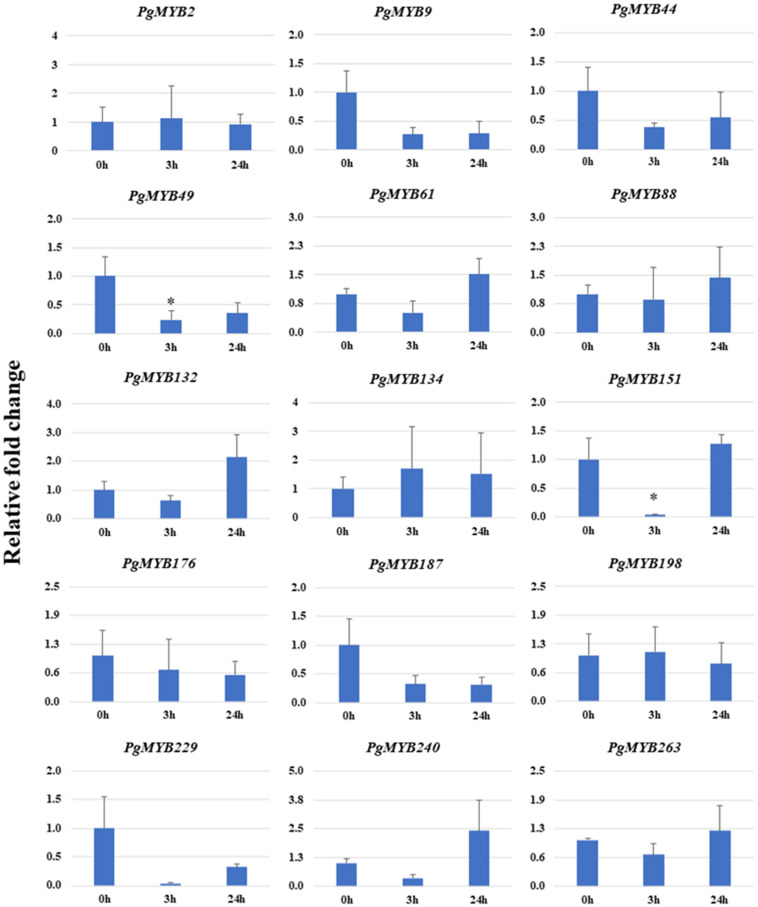
Relative expression analysis of *PgMYB* genes under salinity stress at 0 h, 3 h and 24 h time points. The *X*-axis represents different time points and the *Y*-axis indicates relative expression level. Significant difference in mean indicated by * *p* < 0.05, as obtained by Student’s *t*-test.

In the course of salinity stress, an upregulation of 7 *PgMYBs* and downregulation of 7 *PgMYB* was evinced at early or later time points (Figure 7). The *PgMYBs* showed a differential expression pattern at different time points, such as *PgMYB2* and *PgMYB151* showed changes in expression level at an early time point (3 h). Similarly, the expression level of *PgMYB61*, *PgMYB132*, *PgMYB240*, *PgMYB263* and *PgMYB198* was affected at later time points (24 h). Interestingly, we observed a significant downregulation of *PgMYB9*, *PgMYB49*, *PgMYB187* and *PgMYB229* at both the early (3 h) and late (24 h) time points. Most of the *PgMYBs* showed similar expression pattern in both the transcriptome data and relative expression profiling.

**Figure 8:**
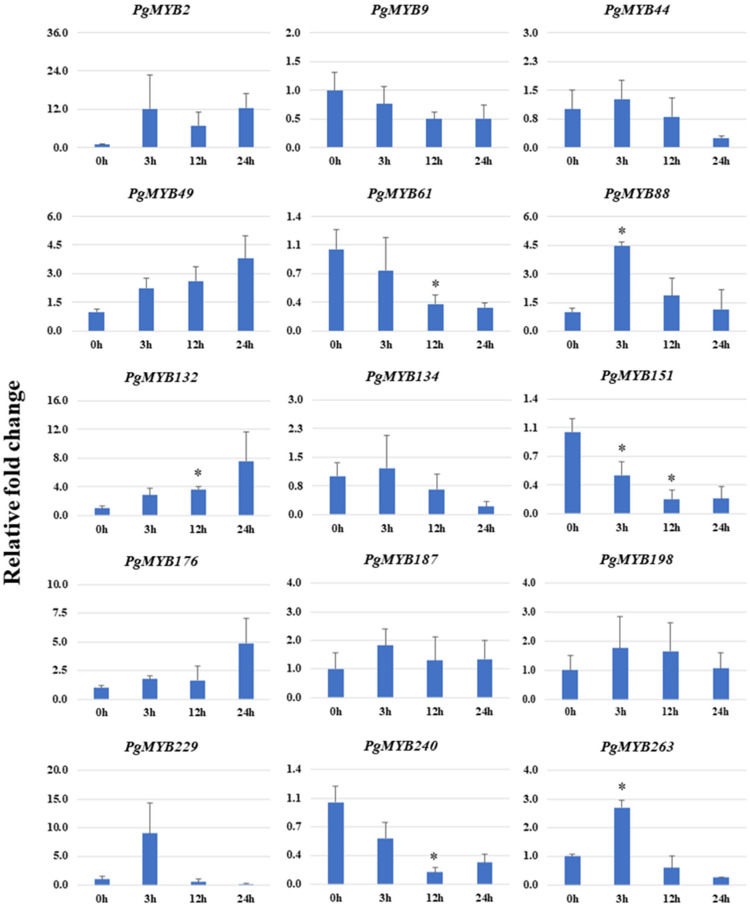
Relative expression analysis of *PgMYB* genes under heat stress at 0 h, 3 h, 12 h and 24 h time points. The *X*-axis represents different time points and the *Y*-axis indicates relative expression level. Significant difference in mean indicated by * *p* < 0.05, as obtained by Student’s *t*-test.

In response to heat stress, nine *PgMYBs* showed upregulation and four *PgMYBs* displayed downregulation (Figure 8). A significant increase in the transcript accumulation of *PgMYB49* and *PgMYB132* was observed, while the expression level of *PgMYB61*, *PgMYB151* and *PgMYB240* was significantly reduced upon heat stress. Interestingly, the transcript level of a few *PgMYB* were comparable to control samples after recovery (at 24 h), suggesting their possible involvement in the heat stress response of pearl millet.

Notably, *PgMYB2*, *PgMYB88* and *PgMYB263* were upregulated under dehydration, salinity and heat stress. Similarly, the transcript accumulation of *PgMYB9* and *PgMYB151* was reduced under the abiotic stress treatments. The differential expression profile under multiple stress conditions indicates their crucial role in abiotic stress responses in pearl millet. Several studies have demonstrated the role of MYB TFs in multiple abiotic stresses [19,52,53].

### 3.9. Relative Expression Analysis of PgMYBs upon Phytohormone Treatments

**Figure 9:**
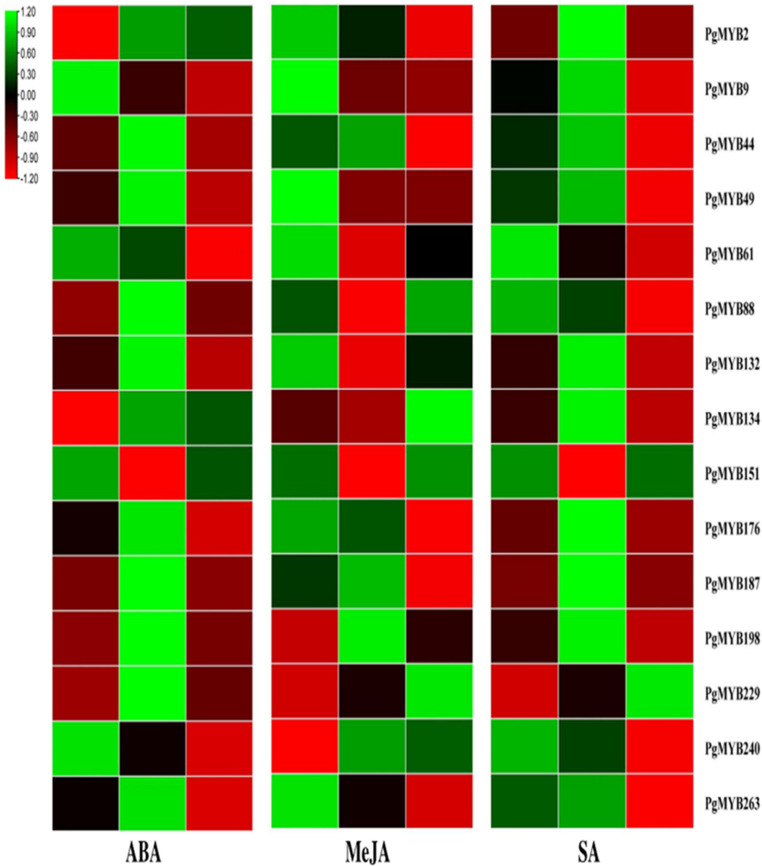
Heat map showing expression profile of *PgMYB* genes upon exogenous phytohormones (ABA, MeJA and SA) treatments at 0 h, 3 h and 24 h time points. The transcript abundance level has been normalized and hierarchically clustered. The heat map was generated using TBtools v0.66831.

Phytohormones are well known for activating the specific signal cascades in response to various environmental stresses [21]. MYB TFs are reported to be involved in phytohormonal signaling pathways under various stress conditions [64]. Thus, in the present study, the expression pattern of *PgMYB* genes was evaluated in response to ABA, MeJA and SA treatment (Figure 9).

The ABA treatment led to the upregulation of *PgMYB2* and *PgMYB134* and downregulation of *PgMYB9*, *PgMYB61* and *PgMYB240* at 3 h and 24 h time points. We have also observed the upregulated expression of eight PgMYBs at the early time point (3 h), while *PgMYB151* showed downregulation at the early time point (3 h) upon ABA treatment. Furthermore, it was shown that the *MYB* family members are involved in the ABA-dependent signal pathway and activate antioxidant enzymes to improve plant stress tolerance [16]. Similarly, under dehydration stress and ABA treatment, *PgMYB2*, *PgMYB44*, *PgMYB49*, *PgMYB88*, *PgMYB134*, *PgMYB187*, *PgMYB198* and *PgMYB229* were induced; meanwhile, *PgMYB9* and *PgMYB151* were downregulated. Therefore, these *PgMYBs* might play an important role in providing drought tolerance through the ABA signaling pathway in pearl millet, though further validation is necessary to confirm these findings.

The significant role of jasmonates and salicylic acid in stress responses has been very well documented [65]. Upon treatment with MeJA, the transcript accumulation of most of the *PgMYBs* was reduced; however, the expression level of *PgMYB134*, *PgMYB198*, *PgMYB229* and *PgMYB240* was increased. Meanwhile, the SA treatment caused downregulation of the majority of the *PgMYB* genes in the pearl millet. The transcript level of *PgMYB229* was upregulated at both the 3 h and 24 h time points. The early upregulated response (at 3 h) of nine *PgMYBs* was also observed in response to an exogenous SA application. However, at the 24 h time point, the expression level of these nine *PgMYBs* was reduced. The differential expression profile of *PgMYBs* upon exogenous phytohormone treatments indicates a possible involvement of the *PgMYBs* in phytohormone stress signaling for implying the plant’s tolerance [66].

Taken together, the differential expression analysis of *PgMYB* genes under abiotic stress and phytohormone treatment indicates their probable involvement in different signaling pathways linked to stress responses in pearl millet.

## 4. Conclusions

In the present study, a total number of 279 putative PgMYBs were identified and distributed unevenly across seven chromosomes of the pearl millet genome. The phylogenetic analysis, motif conservation and gene structure analysis provided insights into the structural diversity of *PgMYBs*. The tandem and segmental duplication events suggest the expansion of the MYB family in pearl millet. In addition, the transcriptome data and relative expression profiling of selected *PgMYBs* in different tissues and upon various stress treatments enabled us to understand their role in pearl millet development and stress responses. The majority of *PgMYB* genes also showed a differential expression profile under abiotic stress, as well as upon phytohormone treatments, which suggest their probable involvement in various phytohormone-signaling pathways for mediating the stress responses in pearl millet. Therefore, our work provides a comprehensive understanding of the MYB family members and their functional role in pearl millet. The information obtained will be useful for understanding the detailed evolutionary progression and functional characterization of candidate PgMYB TFs in plants. Further, the identified candidate *PgMYBs* could contribute significantly towards the development of engineered, multiple stress-tolerant crop plants to ensure better crop productivity.

## Supplementary Materials

The following supporting information can be downloaded at: https://www.mdpi.com/article/10.3390/plants12020355/s1, Figure S1: Membrane-bound MYB transcription factors with a transmembrane motif (TM, blue square) at the C-terminal region; Figure S2: Gene structure analysis of the 279 PgMYB genes of pearl millet. S2a;1-100 PgMYBs, S2b;101-200 PgMYBs, S2c;201-279 PgMYBs. Yellow boxes indicate exons; black lines indicate introns; blue boxes indicate upstream/downstream; Figure S3: In silico expression profiling of *PgMYB* genes under drought (S3a) and salt (S3b) stress using publicly available RNA-seq data; Figure S4: Tissue specific expression analysis of PgMYB genes in Leaf, Stem and Root tissues. Significant difference in mean indicated by * *p* < 0.05, ** *p* < 0.01, as obtained by Student’s t-test; Table S1: Sequence characteristics and chromosomal coordinates of identified 279 PgMYB members in pearl millet; Table S2: List orthologous and paralogous pairs between PgMYB, OsMYB, SiMYB and AtMYB; Table S3: List of primers used in qRT-PCR analysis for selected *PgMYBs*.

## References

[1] Zandalinas S.I., Mittler R. (2022). Plant responses to multifactorial stress combination. New Phytol..

[2] Rivero R.M., Mittler R., Blumwald E., Zandalinas S.I. (2022). Developing climate-resilient crops: Improving plant tolerance to stress combination. Plant J..

[3] Baillo E.H., Kimotho R.N., Zhang Z., Xu P. (2019). Transcription Factors Associated with Abiotic and Biotic Stress Tolerance and Their Potential for Crops Improvement. Genes.

[4] Manna M., Thakur T., Chirom O., Mandlik R., Deshmukh R., Salvi P. (2021). Transcription factors as key molecular target to strengthen the drought stress tolerance in plants. Physiol. Plant..

[5] Wray G.A., Hahn M.W., Abouheif E., Balhoff J.P., Pizer M., Rockman M.V., Romano L.A. (2003). The Evolution of Transcriptional Regulation in Eukaryotes. Mol. Biol. Evol..

[6] Khan S.-A., Li M.-Z., Wang S.-M., Yin H.-J. (2018). Revisiting the Role of Plant Transcription Factors in the Battle against Abiotic Stress. Int. J. Mol. Sci..

[7] Wang H., Wang H., Shao H., Tang X. (2016). Recent Advances in Utilizing Transcription Factors to Improve Plant Abiotic Stress Tolerance by Transgenic Technology. Front. Plant Sci..

[8] Saxena R., Vanga S.K., Wang J., Orsat V., Raghavan V. (2018). Millets for Food Security in the Context of Climate Change: A Review. Sustainability.

[9] Satyavathi C.T., Ambawat S., Khandelwal V., Srivastava R.K. (2021). Pearl Millet: A Climate-Resilient Nutricereal for Mitigating Hidden Hunger and Provide Nutritional Security. Front. Plant Sci..

[10] Srivastava R.K., Yadav O.P., Kaliamoorthy S., Gupta S.K., Serba D.D., Choudhary S., Govindaraj M., Kholová J., Murugesan T., Satyavathi C.T. (2022). Breeding Drought-Tolerant Pearl Millet Using Conventional and Genomic Approaches: Achievements and Prospects. Front. Plant Sci..

[11] Varshney R.K., Shi C., Thudi M., Mariac C., Wallace J., Qi P., Zhang H., Zhao Y., Wang X., Rathore A. (2017). Pearl millet genome sequence provides a resource to improve agronomic traits in arid environments. Nat. Biotechnol..

[12] Ambawat S., Sharma P., Yadav N.R., Yadav R.C. (2013). MYB transcription factor genes as regulators for plant responses: An overview. Physiol. Mol. Biol. Plants.

[13] Jiang C.-K., Rao G.-Y. (2020). Insights into the Diversification and Evolution of R2R3-MYB Transcription Factors in Plants1. Plant Physiol..

[14] Li Z., Peng R., Tian Y., Han H., Xu J., Yao Q. (2016). Genome-Wide Identification and Analysis of the MYB Transcription Factor Superfamily in Solanum lycopersicum. Plant Cell Physiol..

[15] Wang X., Niu Y., Zheng Y. (2021). Multiple Functions of MYB Transcription Factors in Abiotic Stress Responses. Int. J. Mol. Sci..

[16] Jaradat M.R., Feurtado J.A., Huang D., Lu Y., Cutler A.J. (2013). Multiple roles of the transcription factor AtMYBR1/AtMYB44 in ABA signaling, stress responses, and leaf senescence. BMC Plant Biol..

[17] Jung C., Seo J.S., Han S.W., Koo Y.J., Kim C.H., Song S.I., Nahm B.H., Choi Y.D., Cheong J.J. (2008). Overexpression of AtMYB44 enhances stomatal closure to confer abiotic stress tolerance in transgenic Arabidopsis. Plant Physiol..

[18] Vannini C., Locatelli F., Bracale M., Magnani E., Marsoni M., Osnato M., Mattana M., Baldoni E., Coraggio I. (2004). Overexpression of the rice Osmyb4 gene increases chilling and freezing tolerance of *Arabidopsis thaliana* plants. Plant J..

[19] Wei Q., Chen R., Wei X., Liu Y., Zhao S., Yin X., Xie T. (2020). Genome-wide identification of R2R3-MYB family in wheat and functional characteristics of the abiotic stress responsive gene TaMYB344. BMC Genom..

[20] Tang Y., Bao X., Zhi Y., Wu Q., Guo Y., Yin X., Zeng L., Li J., Zhang J., He W. (2019). Overexpression of a MYB Family Gene, OsMYB6, Increases Drought and Salinity Stress Tolerance in Transgenic Rice. Front. Plant Sci..

[21] Verma V., Ravindran P., Kumar P.P. (2016). Plant hormone-mediated regulation of stress responses. BMC Plant Biol..

[22] Ku Y.S., Sintaha M., Cheung M.Y., Lam H.M. (2018). Plant Hormone Signaling Crosstalks between Biotic and Abiotic Stress Responses. Int. J. Mol. Sci..

[23] Vishwakarma K., Upadhyay N., Kumar N., Yadav G., Singh J., Mishra R.K., Kumar V., Verma R., Upadhyay R., Pandey M. (2017). Abscisic acid signaling and abiotic stress tolerance in plants: A review on current knowledge and future prospects. Front. Plant Sci..

[24] Qiu Z., Guo J., Zhu A., Zhang L., Zhang M. (2014). Exogenous jasmonic acid can enhance tolerance of wheat seedlings to salt stress. Ecotoxicol. Environ. Saf..

[25] Wasternack C., Song S. (2017). Jasmonates: Biosynthesis, metabolism, and signaling by proteins activating and repressing transcription. J. Exp. Bot..

[26] Hayat Q., Hayat S., Irfan M., Ahmad A. (2010). Effect of exogenous salicylic acid under changing environment: A review. Environ. Exp. Bot..

[27] Khan M.I.R., Fatma M., Per T.S., Anjum N.A., Khan N.A. (2015). Salicylic acid-induced abiotic stress tolerance and underlying mechanisms in plants. Front. Plant Sci..

[28] Sun M., Huang D., Zhang A., Khan I., Yan H., Wang X., Zhang X., Zhang J., Huang L. (2020). Transcriptome analysis of heat stress and drought stress in pearl millet based on Pacbio full-length transcriptome sequencing. BMC Plant Biol..

[29] Shivhare R., Asif M.H., Lata C. (2020). Comparative transcriptome analysis reveals the genes and pathways involved in terminal drought tolerance in pearl millet. Plant Mol. Biol..

[30] Shinde H., Tanaka K., Dudhate A., Tsugama D., Mine Y., Kamiya T., Gupta S.K., Liu S., Takano T. (2018). Comparative de novo transcriptomic profiling of the salinity stress responsiveness in contrasting pearl millet lines. Environ. Exp. Bot..

[31] Kurata N., Yamazaki Y. (2006). Oryzabase. An integrated biological and genome information database for rice. Plant Physiol..

[32] Yilmaz A., Nishiyama M.Y., Fuentes B.G., Souza G.M., Janies D., Gray J., Grotewold E. (2009). GRASSIUS: A Platform for Comparative Regulatory Genomics across the Grasses. Plant Physiol..

[33] Goodstein D.M., Shu S., Howson R., Neupane R., Hayes R.D., Fazo J., Mitros T., Dirks W., Hellsten U., Putnam N. (2012). Phytozome: A comparative platform for green plant genomics. Nucleic Acids Res..

[34] Finn R.D., Clements J., Eddy S.R. (2011). HMMER web server: Interactive sequence similarity searching. Nucleic Acids Res..

[35] Sonnhammer E.L.L., Eddy S.R., Birney E., Bateman A., Durbin R. (1998). Pfam: Multiple sequence alignments and HMM-profiles of protein domains. Nucleic Acids Res..

[36] Krogh A., Larsson B., von Heijne G., Sonnhammer E.L. (2001). Predicting transmembrane protein topology with a hidden Markov model: Application to complete genomes. J. Mol. Biol..

[37] Horton P., Park K.-J., Obayashi T., Fujita N., Harada H., Adams-Collier C.J., Nakai K. (2007). WoLF PSORT: Protein localization predictor. Nucleic Acids Res..

[38] Hu B., Jin J., Guo A.Y., Zhang H., Luo J., Gao G. (2015). GSDS 2.0: An upgraded gene feature visualization server. Bioinformatics.

[39] Bailey T.L., Boden M., Buske F.A., Frith M., Grant C.E., Clementi L., Ren J., Li W.W., Noble W.S. (2009). MEME SUITE: Tools for motif discovery and searching. Nucleic Acids Res..

[40] Chen C., Chen H., Zhang Y., Thomas H.R., Frank M.H., He Y., Xia R. (2020). TBtools: An Integrative Toolkit Developed for Interactive Analyses of Big Biological Data. Mol. Plant.

[41] Edgar R.C. (2004). MUSCLE: Multiple sequence alignment with high accuracy and high throughput. Nucleic Acids Res..

[42] Kumar S., Stecher G., Tamura K. (2016). MEGA7: Molecular Evolutionary Genetics Analysis Version 7.0 for Bigger Datasets. Mol. Biol. Evol..

[43] Wang Y., Tang H., Debarry J.D., Tan X., Li J., Wang X., Lee T.-h., Jin H., Marler B., Guo H. (2012). MCScanX: A toolkit for detection and evolutionary analysis of gene synteny and collinearity. Nucleic Acids Res..

[44] Siri J.N., Neufeld E., Parkin I.A.P., Sharpe A.G. Using Simulated Annealing to Declutter Genome Visualizations. Proceedings of the FLAIRS Conference.

[45] Rombauts S., Déhais P., Van Montagu M., Rouzé P. (1999). PlantCARE, a plant cis-acting regulatory element database. Nucleic Acids Res..

[46] Langmead B., Salzberg S.L. (2012). Fast gapped-read alignment with Bowtie 2. Nat. Methods.

[47] Grabherr M.G., Haas B.J., Yassour M., Levin J.Z., Thompson D.A., Amit I., Adiconis X., Fan L., Raychowdhury R., Zeng Q. (2011). Full-length transcriptome assembly from RNA-Seq data without a reference genome. Nat. Biotechnol..

[48] Li B., Dewey C.N. (2011). RSEM: Accurate transcript quantification from RNA-Seq data with or without a reference genome. BMC Bioinform..

[49] Chanwala J., Satpati S., Dixit A., Parida A., Giri M.K., Dey N. (2020). Genome-wide identification and expression analysis of WRKY transcription factors in pearl millet (*Pennisetum glaucum*) under dehydration and salinity stress. BMC Genom..

[50] Jha D.K., Chanwala J., Sandeep I.S., Dey N. (2021). Comprehensive identification and expression analysis of GRAS gene family under abiotic stress and phytohormone treatments in Pearl millet. Funct. Plant Biol. FPB.

[51] Reddy P.S., Reddy D.S., Sharma K.K., Bhatnagar-Mathur P., Vadez V. (2015). Cloning and validation of reference genes for normalization of gene expression studies in pearl millet [*Pennisetum glaucum* (L.) R. Br.] by quantitative real-time PCR. Plant Gene.

[52] Katiyar A., Smita S., Lenka S.K., Rajwanshi R., Chinnusamy V., Bansal K.C. (2012). Genome-wide classification and expression analysis of MYB transcription factor families in rice and Arabidopsis. BMC Genom..

[53] Muthamilarasan M., Khandelwal R., Yadav C.B., Bonthala V.S., Khan Y., Prasad M. (2014). Identification and molecular characterization of MYB Transcription Factor Superfamily in C4 model plant foxtail millet (*Setaria italica* L.). PLoS ONE.

[54] Singh V., Kumar N., Dwivedi A.K., Sharma R., Sharma M.K. (2020). Phylogenomic Analysis of R2R3 MYB Transcription Factors in Sorghum and their Role in Conditioning Biofuel Syndrome. Curr. Genom..

[55] Du H., Yang S.S., Liang Z., Feng B.R., Liu L., Huang Y.B., Tang Y.X. (2012). Genome-wide analysis of the MYB transcription factor superfamily in soybean. BMC Plant Biol..

[56] Du H., Feng B.R., Yang S.S., Huang Y.B., Tang Y.X. (2012). The R2R3-MYB transcription factor gene family in maize. PLoS ONE.

[57] Wang D., Yu Y., Liu Z., Li S., Wang Z., Xiang F. (2016). Membrane-bound NAC transcription factors in maize and their contribution to the oxidative stress response. Plant Sci..

[58] De Backer J., Van Breusegem F., De Clercq I. (2022). Proteolytic Activation of Plant Membrane-Bound Transcription Factors. Front. Plant Sci..

[59] Cannon S.B., Mitra A., Baumgarten A., Young N.D., May G. (2004). The roles of segmental and tandem gene duplication in the evolution of large gene families in Arabidopsis thaliana. BMC Plant Biol..

[60] Feng G., Burleigh J.G., Braun E.L., Mei W., Barbazuk W.B. (2017). Evolution of the 3R-MYB Gene Family in Plants. Genome Biol. Evol..

[61] Sheshadri S.A., Nishanth M.J., Simon B. (2016). Stress-Mediated cis-Element Transcription Factor Interactions Interconnecting Primary and Specialized Metabolism in planta. Front. Plant Sci..

[62] Yamaguchi-Shinozaki K., Shinozaki K. (2005). Organization of cis-acting regulatory elements in osmotic- and cold-stress-responsive promoters. Trends Plant Sci..

[63] Ma R., Liu B., Geng X., Ding X., Yan N., Sun X., Wang W., Sun X., Zheng C. (2022). Biological Function and Stress Response Mechanism of MYB Transcription Factor Family Genes. J. Plant Growth Regul..

[64] Li C., Ng C.K.Y., Fan L.-M. (2015). MYB transcription factors, active players in abiotic stress signaling. Environ. Exp. Bot..

[65] Per T.S., Khan M.I.R., Anjum N.A., Masood A., Hussain S.J., Khan N.A. (2018). Jasmonates in plants under abiotic stresses: Crosstalk with other phytohormones matters. Environ. Exp. Bot..

[66] Kapoor B., Kumar P., Sharma R., Kumar A. (2021). Regulatory interactions in phytohormone stress signaling implying plants resistance and resilience mechanisms. J. Plant Biochem. Biotechnol..

