# Supplementary figures and images for: MYB Transcription Factor Family in Pearl Millet: Genome-Wide Identification, Evolutionary Progression and Expression Analysis under Abiotic Stress and Phytohormone Treatments
Source: Plants (Basel). 2023 Jan 12;12(2):355. doi: 10.3390/plants12020355 (PMC9865524; doi:10.3390/plants12020355)

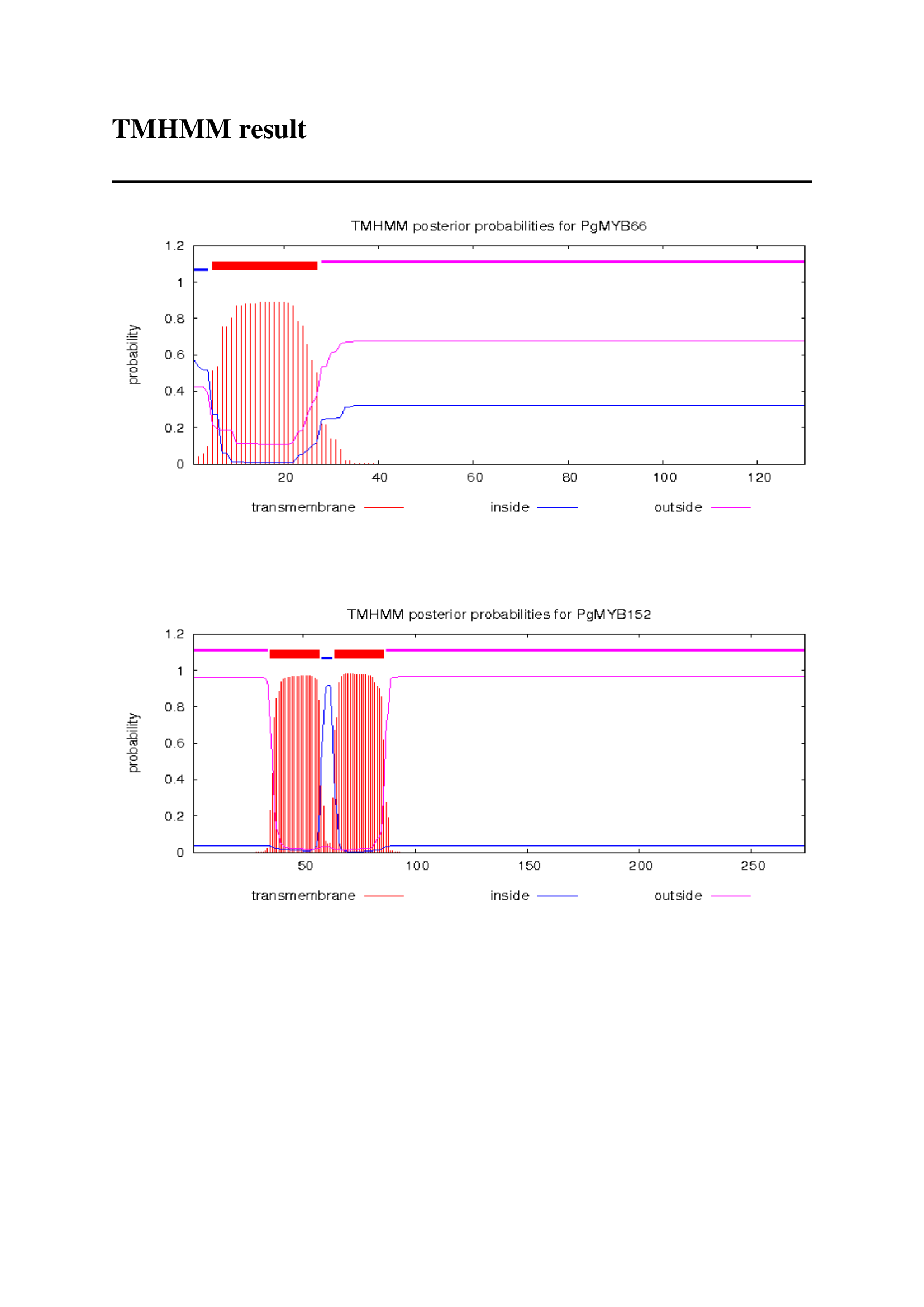

Supplement: Supplementary file 1 [file plants-12-00355-s001.zip › Additional Figure S1.tiff]

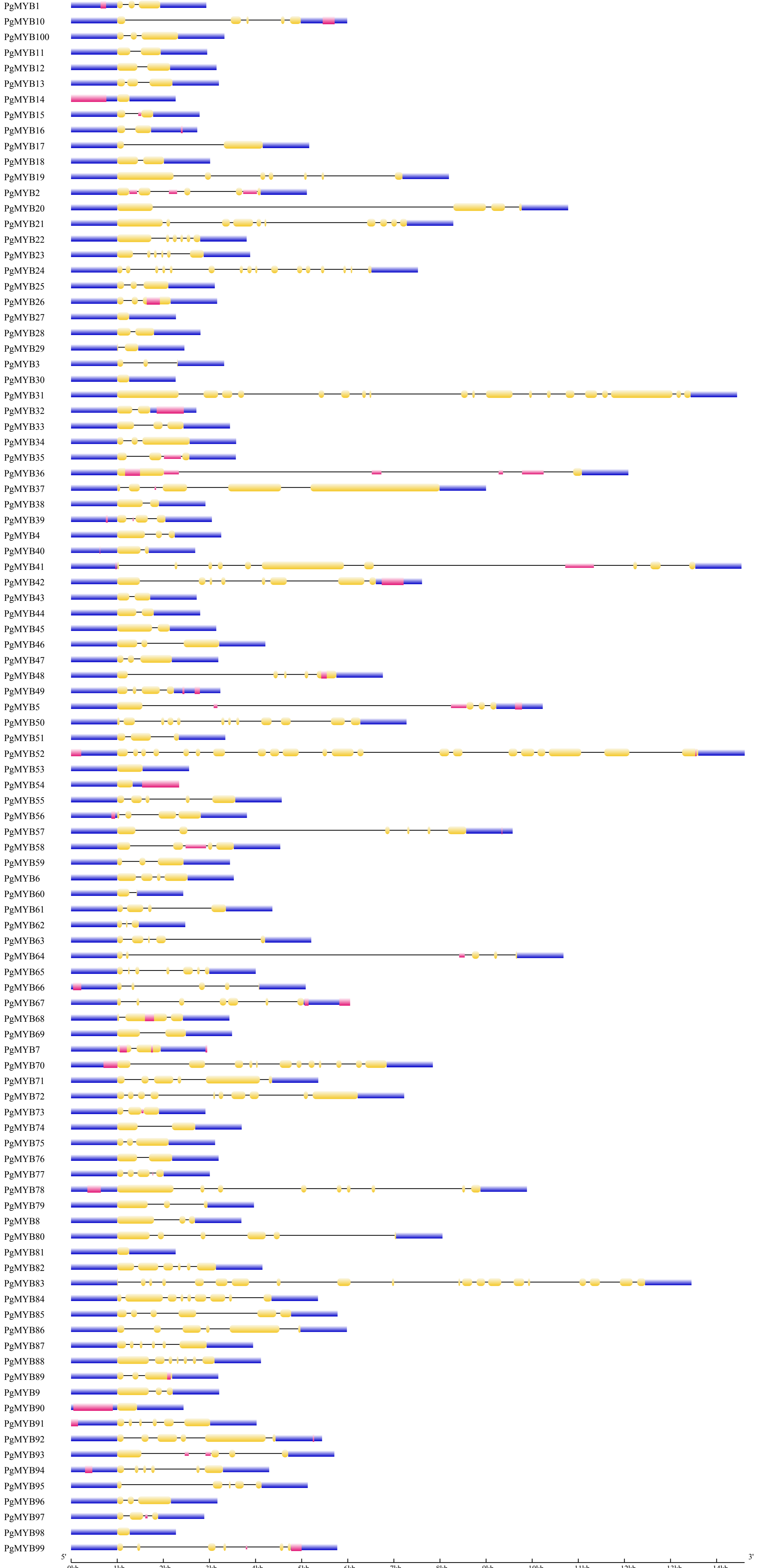

Supplement: Supplementary file 1 [file plants-12-00355-s001.zip › Additional Figure S2a.pdf]

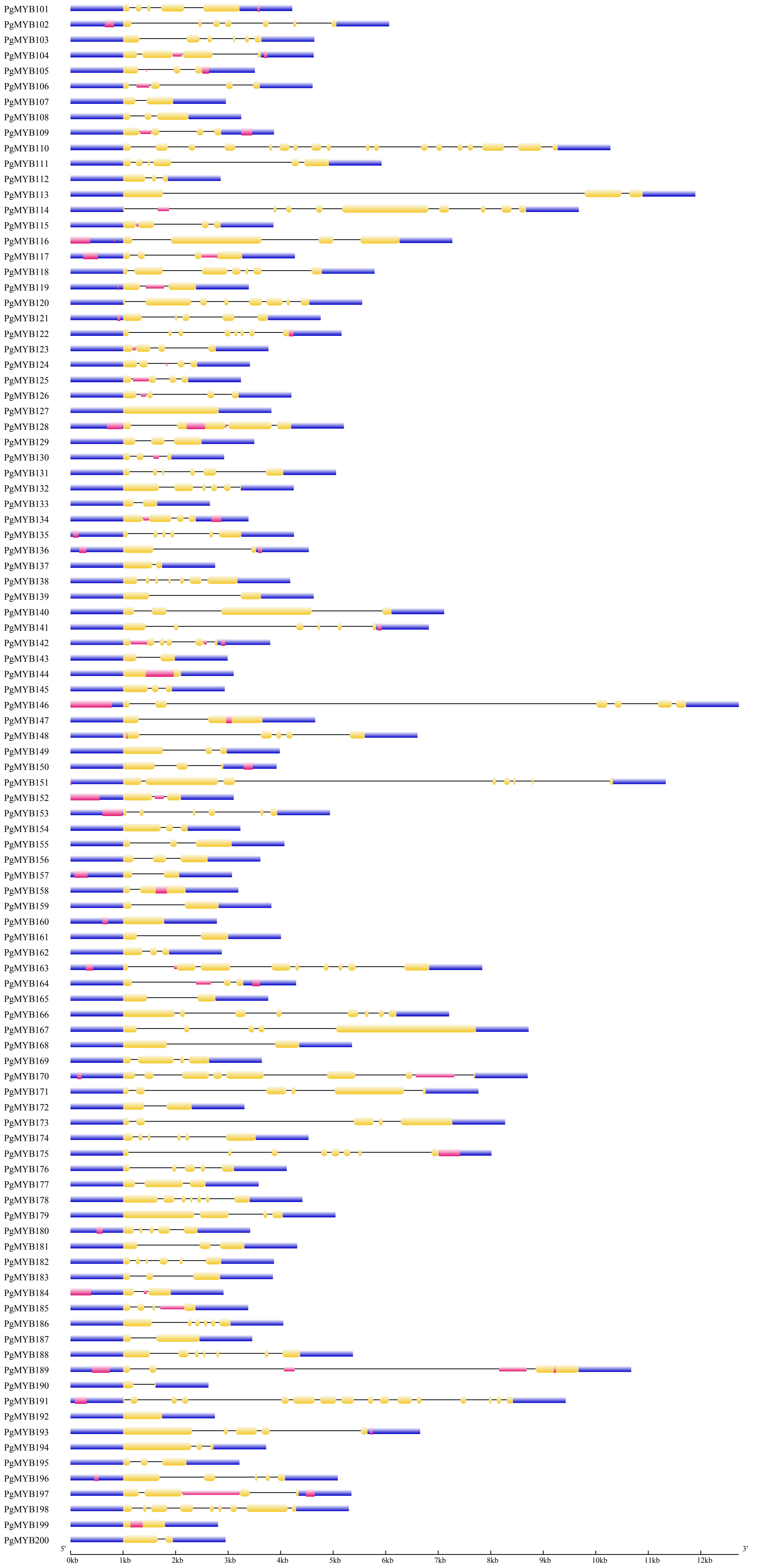

Legend:

- CDS
- upstream/ downstream
- Intron
- NSI

Supplement: Supplementary file 1 [file plants-12-00355-s001.zip › Additional Figure S2b.pdf]

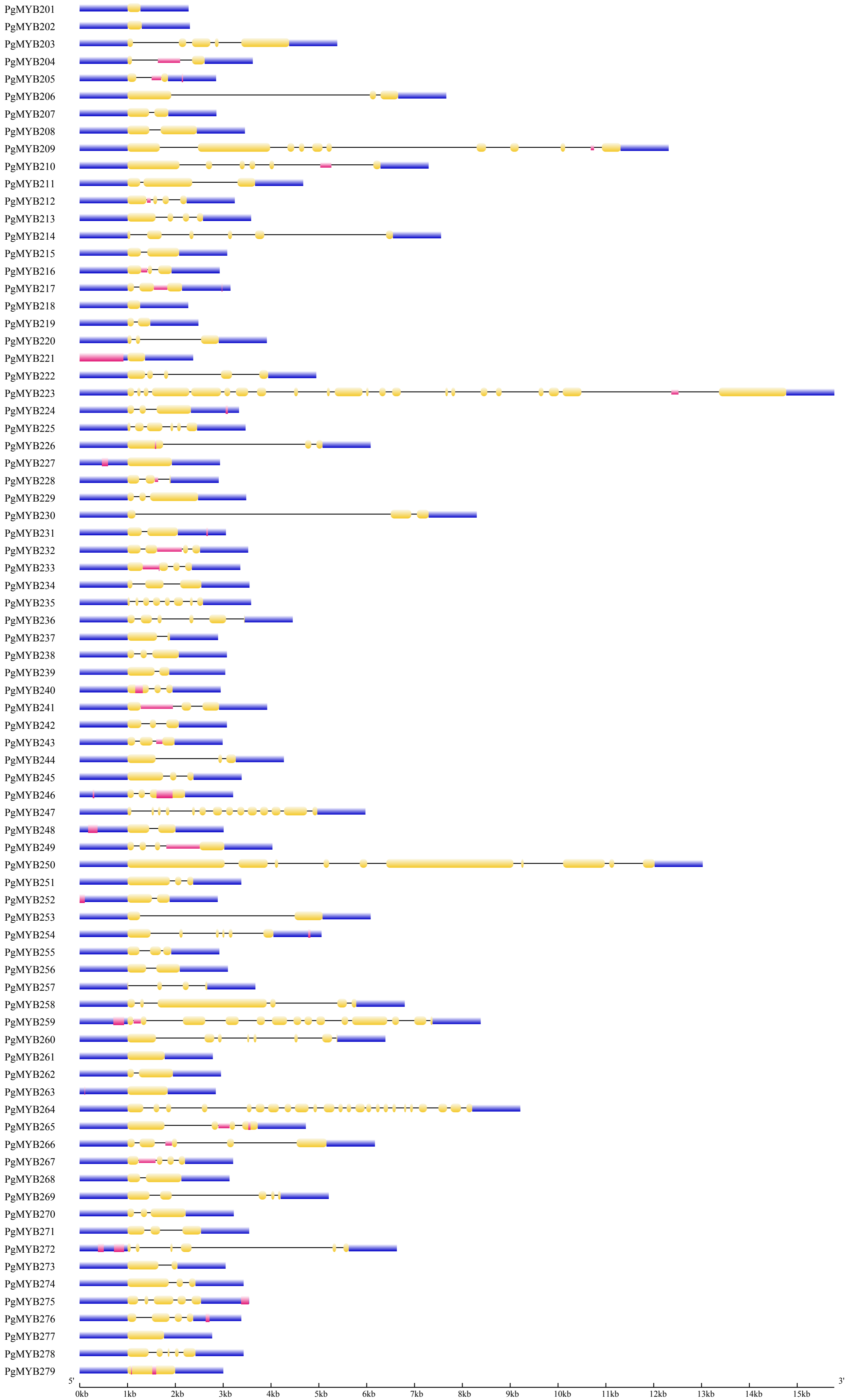

Supplement: Supplementary file 1 [file plants-12-00355-s001.zip › Additional Figure S2c.pdf]

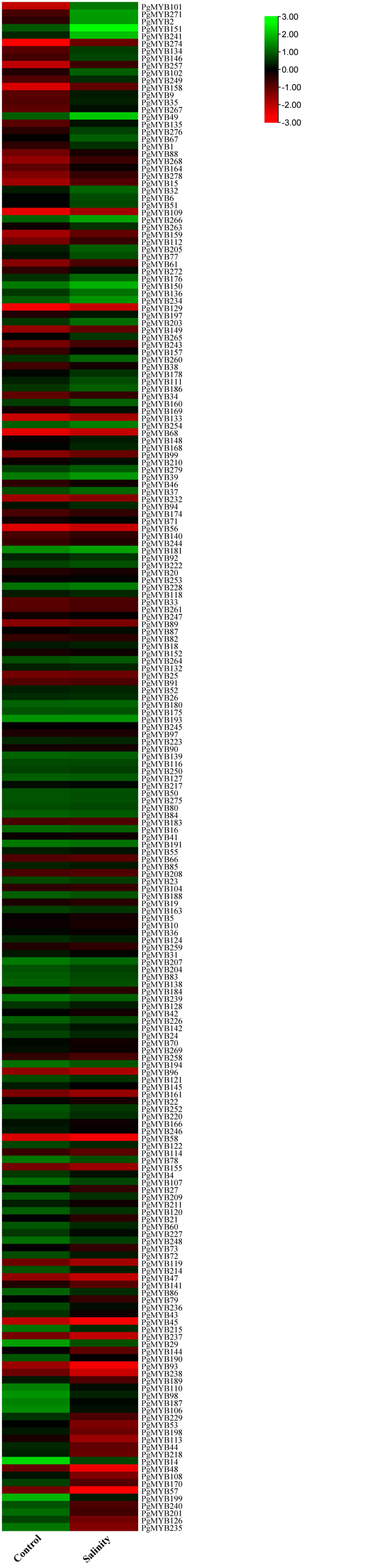

Supplement: Supplementary file 1 [file plants-12-00355-s001.zip › Additional figure S3b.jpg]

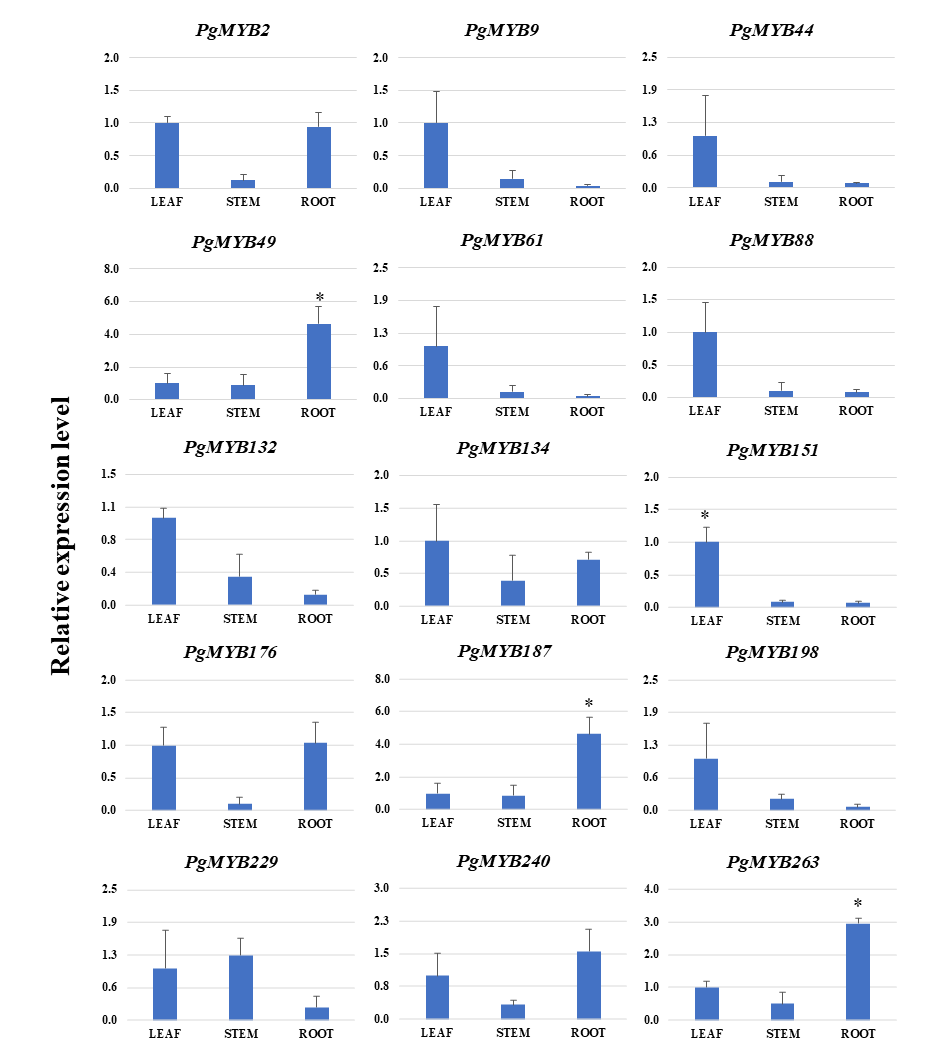

Supplement: Supplementary file 1 [file plants-12-00355-s001.zip › Additional figure s4.tif]
